# How electrons Coulomb repulsion changes graphene band structure

**DOI:** 10.1038/s41598-022-09527-9

**Published:** 2022-03-31

**Authors:** Rostam Moradian, Poorya Rabibeigi

**Affiliations:** 1 Physics Department, Faculty of Science Razi University, Kermanshah, Iran; 2 Nano Science and Nano Technology Research Center, Razi University, Kermanshah, Iran

**Keywords:** Materials science, Nanoscience and technology, Physics

## Abstract

Base on effective medium theory we introduce a multi sites method for calculation of realistic energy bands of strongly correlated systems. We found due to approximated self energy, the density of states that obtained directly by calculated local Green function does not reflects system energy bands truly. By using this method we investigated how electrons repulsion renormalizes graphene bands. Graphene realistic bands calculated in both the dynamical mean field theory (DMFT) and four sites beyond super cell approximation for different repulsions. Our calculated interacting graphene bands illustrate a semi metal to a Mott insulator anti ferromagnetic phase transition at repulsions $u = 2.2 t$ and $u = 0.6 t$ for DMFT and four sites beyond super cell approximation respectively. These values are much less than finite size quantum Monte Carlo calculation prediction. We showed that the graphene bands are very sensitive to electrons repulsions and this phase transition happens at low repulsions in comparison to graphene band width.

## Introduction

Strongly Correlated electrons are responsible for notable properties such as unconventional high $T_{c}$ superconductivity, charge strips in cuprate, magnetites $(A, A')\,{\text{MnO}}_{3}$ where *A* and $A'$ are rare earth and alkali earth elements respectively, nikelates $(A, A')\, {\text {NiO}}_{3}$, Mott transitions and quantum critical phenomena^1–6^. Transition metal oxides are a class of materials that electron–electron Coulomb repulsion in their valance d-wave orbitals is strong. Conventional single electron band theories break down for these systems. Although theoretical calculations of these systems properties are hard but their applications is wide^7^. How electron–electron Coulomb repulsion modifies electronic band structure of these systems is a big change in strongly correlated systems. Usually in the calculations of physical quantities of such systems two different methods finite size approximation^8–15^ and effective medium theory are using^16–25^. For a honeycomb graphene lattice by using Hubbard model with on site electrons repulsion and a finite size quantum Monte Carlo calculations for $u/t > 4.5$ a non magnetic semi metallic to an anti ferromagnetic insulator phase transition predicted^26–28^.

In the effective medium theories the electron–electron interaction effects replaces by an effective medium that identifies by a self energy $\Sigma (\mathbf{k}, E)$. However self energy changes non interacting band structure $E^{0}_\mathbf{k}$ to renormalized band structure $E_\mathbf{k}$. In general for most of these systems, self energy could not calculated exactly. One of main problem in condensed matter physics is choosing type of approximation in the self energy calculations. For weak interaction Hartree Fock approximation that is a mean field approximation widely used^29^. Dynamical mean field theory (DMFT) is a single site approximation widely used for any interaction strength *u*^16,17^. In the DMFT inter sites correlation is neglected. Multi sites, *Nc*, dynamical cluster approximation (DCA) for including multi sites correlation is introduced^18–21^. Although they claimed that DCA recovers exact self energy $\Sigma (\mathbf{k}, E)$ in the limit of $lim_{Nc\rightarrow {\mathcal {N}}}$ but their inverse Fourier transform definition of K-space Green function and self energy dose not lead to real space Dyson equation^22^. The DCA coarse grained self energies $\Sigma (\mathbf{K}_{n};E)$ are step functions in the first Brillouin zone that are discontinuous at their grain boundaries. Effective medium super cell approximation (EMSCA)^23–25^ for disorder systems introduced. In the EMSCA relation between real space and k-space grained self energies are $\Sigma (I,J;E)=\frac{1}{Nc}\sum _{\mathbf{K}_{n}}\Sigma (\mathbf{K}_{n}, E) e^{-i\mathbf{K}_{n}.\mathbf{r}_{IJ}}$ and $\Sigma (\mathbf{K}_{n};E)=\frac{1}{Nc}\sum _{IJ}\Sigma (I, J, E) e^{i\mathbf{K}_{n}.\mathbf{r}_{IJ}}$ where in the limit $Nc\rightarrow \ {\mathcal {N}}$ both of them recovers exact k-space and real space self energies. For eliminating discontinuities of k-space self energies $\Sigma (\mathbf{K}_{n}, E)$ in the first Brillouin zone we introduce another relation $\Sigma (\mathbf{k};E)=\frac{1}{Nc}\sum _{IJ}\Sigma (I, J, E) e^{i\mathbf{k}.\mathbf{r}_{IJ}}=\frac{1}{Nc^{2}}\sum _{IJ}\sum _{\mathbf{K}_{n}}\Sigma (\mathbf{K}_{n}; E) e^{i(\mathbf{k}-\mathbf{K}_{n}). \mathbf{r}_{IJ}}$ which in the limit $Nc\rightarrow \ {\mathcal {N}}$ recovers both DMFT and exact self energies. Another main problem of approximated self energies is creating fake electronic states that should be eliminated. This problem solved by equating Dirac delta function shape of k-space density of states at its *m*th maximum with *m*th band energy $E=E_{m\mathbf{k}}$. Note that number of k-space density of states maximums in terms of energy identifies number of contributed bands. We apply this method to a correlated graphene system. In contrast to the finite size quantum Monte Carlo predictions^26–28^, our results show that graphene bands are very sensitive to electrons Coulomb repulsion, hence a semi metal to a Mott insulator phase transition occurs at very low repulsions in comparison to graphene band width.

## Model Hamiltonian and solution of equation of motion

We start our investigation by a Hubbard model for a strongly correlated system which is given by,

1 $$\begin{aligned} H= & {} -\sum _{ij\sigma \sigma }t^{\alpha \beta }_{ij}{c^{\alpha }_{i\sigma }}^{\dagger }c^{\beta }_{j\sigma }+\sum _{i\alpha }u {\hat{n}}^{\alpha }_{i\uparrow }{\hat{n}}^{\alpha }_{i\downarrow }-\sum _{i\sigma } \mu { c^{\alpha }_{i\sigma }}^{\dagger }c^{\alpha }_{i\sigma }, \end{aligned}$$

where ${ c^{\alpha }_{i\sigma }}^{\dagger }$ ($c^{\alpha }_{i\sigma }$) is the creation (annihilation) operator of an electron with spin $\sigma$ on $\alpha$ sub site of lattice site *i* and ${\hat{n}}^{\alpha }_{i\sigma }={c^{\alpha }_{i\sigma }}^{\dagger }c^{\alpha }_{i\sigma }$ is the electrons number operator. $t^{\alpha \sigma \beta \sigma }_{ij}$ are the hopping integrals between $\alpha$ sub site of *i* and $\beta$ sub site of *j* lattice sites. $\mu$ is the chemical potential. Nearest neighbors hoping integral is $t=2.75 {\text {eV}}$

In the imaginary time $\tau$, the equation of motion for electrons corresponding to the above Hamiltonian, Eq. (1), is given by,

2 $$\begin{aligned} \sum _{l\gamma }\left( (-\mathbf{I}\frac{\partial }{\partial \tau }+\mu \mathbf{I})\delta _{il}\delta _{\gamma \alpha }+\mathbf{t}^{\alpha \gamma }_{il}\right) \mathbf{G}^{\gamma \beta }(l,j;\tau )-\sum _{\gamma }\mathbf{G}_{2}(i\alpha ,i\gamma ,j\beta ;\tau ) =\delta (\tau )\delta _{ij}\delta _{\alpha \beta }{} \mathbf{I}, \end{aligned}$$

where $\mathbf {I}$ is a spin space $2\times 2$ unit matrix, $\mathbf{t}^{\alpha \beta }_{ij}=t^{\alpha \uparrow \beta \uparrow }_{ij}{} \mathbf{I}$ and two particle Green function $\mathbf{G}_{2}(i,j;\tau )$ defined by

3 $$\begin{aligned} \mathbf{G}_{2}(i\alpha ,i\gamma ,j\beta ;\tau ) =\left( \begin{array}{ll} u\langle \tau c^{\alpha }_{i \uparrow }(\tau ){c^{\gamma }_{i\downarrow }(\tau )}^{\dagger }c^{\gamma }_{i\downarrow }(\tau ){c^{\beta }_{j \uparrow }}^{\dagger }(0)\rangle &{} u\langle \tau c^{\alpha }_{i \uparrow }(\tau ){c^{\gamma }_{i\downarrow }}^{\dagger }(\tau )c^{\gamma }_{i\downarrow }(\tau ){c^{\beta }_{j \downarrow }}^{\dagger }(0)\rangle \\ u\langle \tau c^{\alpha }_{i \downarrow }(\tau ){c^{\gamma }_{i\uparrow }}^{\dagger }(\tau )c^{\gamma }_{i\uparrow }(\tau ){c^{\beta }_{j \uparrow }}^{\dagger }(0)\rangle &{} u\langle \tau c^{\alpha }_{i \downarrow }(\tau ){c^{\gamma }_{i\uparrow }}^{\dagger }(\tau )c^{\gamma }_{i\uparrow }(\tau ){c^{\beta }_{j \downarrow }}^{\dagger }(0)\rangle \end{array}\right) . \end{aligned}$$

The single particle equation of motion corresponding to Eq. (2) in the effective medium theory is given by

4 $$\begin{aligned} \sum _{l\gamma }\left( (-\mathbf{I}\frac{\partial }{\partial \tau }+\mu \mathbf{I})\delta _{il}\delta _{\gamma \alpha }+\mathbf{t}^{\alpha \gamma }_{il}\right) {\bar{\mathbf{G}}}^{\gamma \beta }(l,j;\tau )-\sum _{l\gamma }{\varvec{\Sigma }}^{\alpha \gamma }(i,l;\tau ){\bar{\mathbf{G}}}^{\gamma \beta }(l,j;\tau ) =\delta (\tau )\delta _{ij}\delta _{\alpha \beta }{} \mathbf{I}, \end{aligned}$$

where self energy matrix ${\varvec{\Sigma }}^{\alpha \gamma }(i,l;\tau )$ defined by

5 $$\begin{aligned} \sum _{\gamma }\langle \mathbf{G}_{2}(i\alpha ,i\gamma ,j\beta ;\tau )\rangle =\sum _{l\gamma }{\varvec{\Sigma }}^{\alpha \gamma }(i,l;\tau ){\bar{\mathbf{G}}}^{\gamma \beta }(l,j;\tau ). \end{aligned}$$

In the real and spin spaces the following relation between interacting single particle Green function $\mathbf{G}(\tau )$, average single particle Green function ${\bar{\mathbf{G}}}(\tau )$ and two particle Green function $\mathbf{G}_{2}(\tau )$ obtains from Eqs. (2) and (5)

6 $$\begin{aligned} \mathbf{G}(\tau )={\bar{\mathbf{G}}}(\tau ) +{\bar{\mathbf{G}}}(\tau )\left( \mathbf{G}_{2}(\tau )- {\varvec{\Sigma }}(\tau )\mathbf{G}(\tau )\right) . \end{aligned}$$

**Figure 1:**
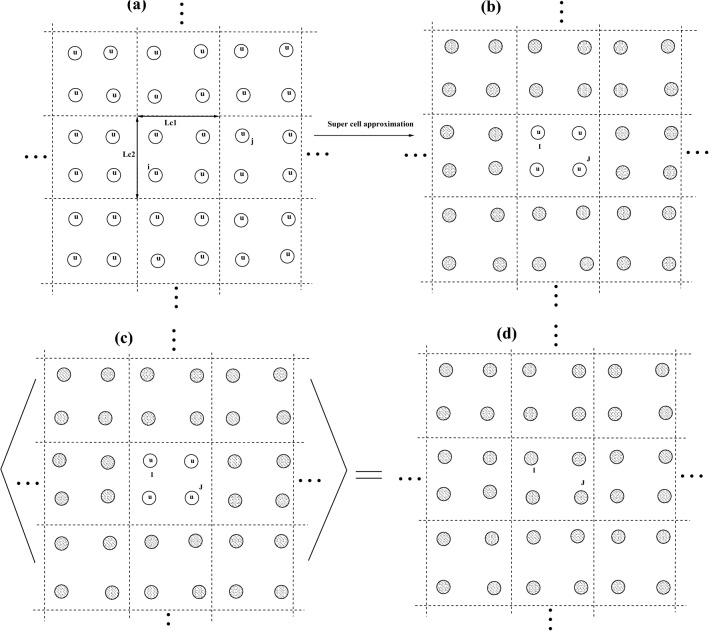
Shows (**a–d**) interacting system, taking average except on impurity super cell, average of impurity super cell respectively.

For solving Eq. (6) we introduce effective medium super cell approximation (EMSCA). In this approximation the interacting system divides into super cells with original lattice symmetries. Process steps of EMSCA illustrated in Fig. 1. First we keep one interacting super cell called impurity super cell that its sites denotes by $\{I, J\in sc\}$ and replace all other super cells by effective medium super cells that schematically illustrated in Fig. 1b. Then by taking average over impurity super cell we obtain average super cell physical properties that illustrated in Fig. 1c.

By applying EMSCA to the interacting system, Eq. (6) reduces to^25^

7 $$\begin{aligned} \mathbf{G}^{imp}_{sc}(\tau )={\bar{\mathbf{G}}}_{sc}(\tau ) +{\bar{\mathbf{G}}}_{sc}(\tau )\left( \mathbf{G}^{imp}_{2 sc}(\tau )- {\varvec{\Sigma }}_{sc}(\tau )\mathbf{G}^{imp}_{sc}(\tau )\right) . \end{aligned}$$

Equation (7) could be written as

8 $$\begin{aligned} {{\bar{\mathbf{G}}}_{sc}(\tau )}^{-1}+{\varvec{\Sigma }}_{sc}(\tau )=( \mathbf{G}^{imp}_{2 sc}(\tau ) +\mathbf{I}_{2Nc\times 2Nc}) {\mathbf{G}^{imp}_{sc}(\tau )}^{-1}={\varvec{{\mathcal {G}}}_{sc}(\tau )}^{-1} . \end{aligned}$$

Equation (8) could be separated into two following equations

9 $$\begin{aligned} {{\bar{\mathbf{G}}}_{sc}}(\tau )={\varvec{{\mathcal {G}}}}_{sc}(\tau )+{\varvec{{\mathcal {G}}}}_{sc}(\tau ) {\varvec{\Sigma }}_{sc}(\tau ){{\bar{\mathbf{G}}}_{sc}}(\tau ), \end{aligned}$$

and

10 $$\begin{aligned} \mathbf{G}^{imp}_{sc}(\tau )={\varvec{{\mathcal {G}}}}_{sc}(\tau )+{\varvec{{\mathcal {G}}}}_{sc}(\tau )\mathbf{G}^{imp}_{2sc}(\tau ), \end{aligned}$$

where ${\varvec{{\mathcal {G}}}}_{sc}(\tau )$ is called super cell cavity Green function with no interaction on its sites. Equations (9) and (10) imply that our interacting calculations reduced to a super cell. Super cell average of Eq. (10) leads to Eq. (9) where

11 $$\begin{aligned} \left\langle \mathbf{G}^{imp}_{2sc}(\tau )\rangle ={\varvec{\Sigma }}_{sc}{\bar{\mathbf{G}}}^{\sigma \sigma }_{sc}(\tau ),\;\langle \mathbf{G}^{imp\;\sigma \sigma }_{sc}(\tau ) \right\rangle = {\bar{\mathbf{G}}}^{\sigma \sigma }_{sc}(\tau ). \end{aligned}$$

Another way for deriving Eqs. (9) and (10) is applying effective medium super cell approximation (EMSCA) to the interacting system total action, *S*, in the following partition function *Z*,

12 $$\begin{aligned} Z=\left\langle e^{-\beta H} \right\rangle =\sum _{l\sigma ^{''}}<\sigma ^{''} l| e^{-\beta H} |l\sigma ^{''}>, \end{aligned}$$

where $\{|l\sigma ^{''}>\}$ are system eigen states. Partition function Eq. (12) could be written as

13 $$\begin{aligned} Z=\sum _{l\sigma ^{''}}<\sigma ^{''} l| e^{-\beta H} |l\sigma ^{''}>= \int {{\mathcal {D}}}\Psi {{\mathcal {D}}}{{\bar{\Psi }}} e^{-S}, \end{aligned}$$

where ${{\mathcal {D}}}\Psi =\Pi ^{{\mathcal {N}}}_{i=1}d\psi _{i\sigma }$, $d\psi _{i\sigma }=lim_{L\rightarrow \infty }\Pi ^{L}_{m=1}d\psi _{i\sigma }(\tau _{m})$, ${{\mathcal {D}}}\bar{\Psi }=\Pi ^{{\mathcal {N}}}_{i=1}d{{\bar{\psi }}}_{i\sigma }$, $d{{\bar{\psi }}}_{i\sigma }=lim_{L\rightarrow \infty }\Pi ^{L}_{m=1}d{{\bar{\psi }}}_{i\sigma }(\tau _{m})$ and *S* is action defined by

14 $$\begin{aligned} S=\sum _{i\sigma j\sigma ^{'}} \int ^{\beta }_{0} d\tau {\bar{\psi }}_{i\sigma }(\tau )\delta _{\sigma \sigma ^{'}}\left( (-\frac{\partial }{\partial \tau }+\mu )\delta _{ij}+t^{\sigma \sigma }_{ij}\right) \psi _{j\sigma ^{'}}(\tau )+S_{int}, \end{aligned}$$

in which interaction action $S_{int}$ is

15 $$\begin{aligned} S_{int}= & {} -\sum _{i} \int ^{\beta }_{0} d\tau {\bar{\psi }}_{i\uparrow }(\tau ) {\psi }_{i\uparrow }(\tau ) u{\bar{\psi }}_{i\downarrow }(\tau )\psi _{i\downarrow }(\tau ) \end{aligned}$$

where $\psi _{i\sigma }$ and ${{\bar{\psi }}}_{i\sigma }$ are Grassmann variables that obey Grassmann algebra. By substitution $\psi _{j\sigma }(\tau )=\frac{1}{\beta } \sum _{\omega _{n}} e^{-i\omega _{n}\tau }\psi (i\omega _{n})$ in Eq. (14) we have

16 $$\begin{aligned} \delta _{\sigma \sigma ^{'}}\left( (-\frac{\partial }{\partial \tau }+\mu )\delta _{ij}+t^{\sigma \sigma }_{ij}\right) \psi _{j\sigma ^{'}}(\tau )= & {} \int d\tau ^{'}\delta _{\sigma \sigma ^{'}}\frac{1}{\beta }\sum _{\omega _{n}}\left( (i\omega _{n}+\mu )\delta _{ij}+t^{\sigma \sigma }_{ij}\right) e^{i\omega _{n}(\tau -\tau ^{'})}\psi _{j\sigma ^{'}}(\tau ^{'}) \nonumber \\= & {} \int d\tau ^{'} \left( {\mathbf{G}^{0}}^{-1}(\tau -\tau ^{'}) \right) _{i\sigma j\sigma ^{'}} \psi _{j\sigma ^{'}}(\tau ^{'}), \end{aligned}$$

where

17 $$\begin{aligned} \left( {\mathbf{G}^{0}}^{-1}(\tau -\tau ^{'}) \right) _{i\sigma j\sigma ^{'}}= \frac{\delta _{\sigma \sigma ^{'}}}{\beta }\sum _{\omega _{n}}\left( (i\omega _{n}+\mu )\delta _{ij}+t^{\sigma \sigma }_{ij}\right) e^{-i\omega _{n}(\tau -\tau ^{'})}. \end{aligned}$$

Relation between non interacting Green function $\mathbf{G}^{0}(i\omega _{n})$ and effective medium average Green function ${\bar{\mathbf{G}}}(i\omega _{n})$ is the following Dyson equation

18 $$\begin{aligned} (\mathbf{G}^{0}(i\omega _{n}))^{-1}=({\bar{\mathbf{G}}}(i\omega _{n}))^{-1} + {\varvec{\Sigma }}(i\omega _{n}). \end{aligned}$$

By imaginary time Fourier transform of Dyson Eq. (18) we have

19 $$\begin{aligned} \frac{1}{\beta }\sum _{\omega _{n}}(\mathbf{G}^{0}(i\omega _{n}))^{-1} e^{-i\omega _{n}(\tau -\tau ^{'})}=\frac{1}{\beta }\sum _{\omega _{n}}({\bar{\mathbf{G}}}(i\omega _{n}))^{-1}e^{-i\omega _{n}(\tau -\tau ^{'})} + \frac{1}{\beta }\sum _{\omega _{n}}{\varvec{\Sigma }}(i\omega _{n}) e^{-i\omega _{n}(\tau -\tau ^{'})}. \end{aligned}$$

Equation (19) denotes by

20 $$\begin{aligned} (\mathbf{G}^{0}(i\omega _{n}))^{-1} (\tau -\tau ^{'})=({\bar{\mathbf{G}}}(i\omega _{n}))^{-1}(\tau -\tau ^{'}) + {\varvec{\Sigma }}(\tau -\tau ^{'}). \end{aligned}$$

By substitution Eq. (20) in the action Eq. (14) we have

21 $$\begin{aligned} S=\sum _{i\sigma j\sigma ^{'}} \int ^{\beta }_{0}\int ^{\beta }_{0}d\tau ^{'} d\tau {\bar{\psi }}_{i\sigma }(\tau ) \left( ({\bar{\mathbf{G}}}(i\omega _{n}))^{-1}(\tau -\tau ^{'}) + {\varvec{\Sigma }}(\tau -\tau ^{'}) \right) _{i\sigma j\sigma ^{'}}\psi _{j\sigma ^{'}}(\tau ^{'})+S_{int}. \end{aligned}$$

Now we apply EMSCA that keeping interaction in the central super cell $i,j\in \{I,J\}$ and replaces others by effective medium super cell self energies ${\varvec{\Sigma }}_{sc}(\tau -\tau ^{'})$

22 $$\begin{aligned} S_{EMSCA}= & {} \sum _{I\sigma J\sigma ^{'}} \int ^{\beta }_{0} d\tau \int ^{\beta }_{0}d\tau ^{'} {\bar{\psi }}_{I\sigma }(\tau ) \left( \left( \varvec{{\mathcal {G}}}_{sc}(i\omega _{n})\right) ^{-1}(\tau -\tau ^{'})\right) _{I\sigma J\sigma ^{'}}\psi _{J\sigma ^{'}}(\tau ^{'})\nonumber \\&- \sum _{I} \int ^{\beta }_{0} d\tau {\bar{\psi }}_{I\uparrow }(\tau ) {\psi }_{I\uparrow }(\tau ) U{\bar{\psi }}_{I\downarrow }(\tau )\psi _{I\downarrow }(\tau )\nonumber \\&+\sum _{i\sigma j\sigma ^{'}, i,j\not \in \{I,J\}} \int ^{\beta }_{0} \int ^{\beta }_{0} d\tau ^{'}d\tau {\bar{\psi }}_{i\sigma }(\tau ) \left( ({\bar{\mathbf{G}}}_{sc}(i\omega _{n}))^{-1}(\tau -\tau ^{'}) \right) _{i\sigma j\sigma ^{'}}\psi _{j\sigma ^{'}}(\tau ^{'}), \end{aligned}$$

where imaginary time real space cavity Green function matrix $\left( \varvec{{\mathcal {G}}}(i\omega _{n})\right) ^{-1}(\tau -\tau ^{'})$ defined by

23 $$\begin{aligned} \left( \varvec{{\mathcal {G}}}_{sc}(i\omega _{n})\right) ^{-1}(\tau -\tau ^{'}) =({\bar{\mathbf{G}}}_{sc}(i\omega _{n}))^{-1}(\tau -\tau ^{'}) + {\varvec{\Sigma }}_{sc}(\tau -\tau ^{'}) . \end{aligned}$$

By inverse imaginary Fourier transform of Eq. (23) we have

24 $$\begin{aligned} {\bar{\mathbf{G}}}_{sc}(i\omega _{n}) =\varvec{{\mathcal {G}}}_{sc}(i\omega _{n}) + \varvec{{\mathcal {G}}}_{sc}(i\omega _{n}){\varvec{\Sigma }}_{sc}(i\omega _{n}) {\bar{\mathbf{G}}}_{sc}(i\omega _{n}). \end{aligned}$$

By imaginary time Fourier transform of first and third terms of right hand side of Eq. (22) we have

25 $$\begin{aligned} S_{EMSCA}=S_{sc}+S_{medium}, \end{aligned}$$

where the central super cell interacting action is

26 $$\begin{aligned} S_{sc}=\frac{1}{\beta }\sum _{\omega _{n}}\sum _{I\sigma J\sigma ^{'}} {\bar{\psi }}_{I\sigma }(i\omega _{n}) \left( \left( \varvec{{\mathcal {G}}}_{sc}(i\omega _{n})\right) ^{-1}\right) _{I\sigma J\sigma ^{'}}\psi _{J\sigma ^{'}}(i\omega _{n})-\sum _{I} \int ^{\beta }_{0} d\tau {\bar{\psi }}_{I\uparrow }(\tau ) {\psi }_{I\uparrow }(\tau ) u {\bar{\psi }}_{I\downarrow }(\tau )\psi _{I\downarrow }(\tau ), \end{aligned}$$

and effective medium action is defined by

27 $$\begin{aligned} S_{medium}=\frac{1}{\beta }\sum _{\omega _{n}}\sum _{i\sigma j\sigma ^{'}, i,j\not \in \{I,J\}} {\bar{\psi }}_{i\sigma }(i\omega _{n}) \left( \left( {\bar{\mathbf{G}}}(i\omega _{n})\right) ^{-1}\right) _{i\sigma j\sigma ^{'}}\psi _{j\sigma ^{'}}(i\omega _{n}). \end{aligned}$$

So partition function in this approximation is product of central impurity super cell $Z_{sc}=e^{S_{sc}}$ and medium $Z_{medium}= e^{S_{medium}}$ where

28 $$\begin{aligned} Z=Z_{medium} Z_{sc}. \end{aligned}$$

To complete loop of super cell single particle average Green function $\mathbf{G}_{sc}(i\omega _{n})$ calculations by Eqs. (9), (10) and (11) methods such as exact diagonalization, quantum Monte Carlo could be used to obtain super cell interacting action $S_{sc}$ and $\mathbf{G}^{imp}_{2sc}(\tau )$ in terms of super cell single particle impurity Green function $\mathbf{G}^{imp}_{sc}(\tau )$ and super cell self energies ${\varvec{\Sigma }}_{sc}(\tau )$. Here we use quantum Monte Carlo method.

### Quantum Monte Carlo method for calculation ${G}^{imp}_{2sc}(\tau )$

To solve Eq. (10) we should find the super cell impurity Green function $\mathbf{G}^{imp}_{2sc}(\tau )$ in terms of super cell single particle Green function $\mathbf{G}^{imp}_{sc}(\tau )$. To do this here we use following Hubbard–Stratonovich transformation^17,21^. By dividing imaginary time $\tau \in [0,\beta ]$ into *M* small portions $\Delta \tau =\frac{\beta }{M}$ and using Hubbard–Stratonovich transformation it is possible to convert action $S_{sc}$ to Ising like fields^17,21^ hence impurity super cell two particle Green function $\mathbf{G}^{imp}_{2sc}(\tau )$ into production of an Ising like fields^17,21^ and $\mathbf{G}^{imp}_{sc}(\tau ^{'})$

29 $$\begin{aligned} \mathbf{G}^{imp}_{sc}(i\omega _{n})= \varvec{{\mathcal {G}}}_{sc}(i\omega _{n})+\varvec{{\mathcal {G}}}_{sc}(i\omega _{n}){\mathcal {V}}_{\{s_{0}\}} \mathbf{G}^{imp}_{sc}(i\omega _{n}), \end{aligned}$$

where

30 $$\begin{aligned} {{\mathcal {V}}}_{\{s_{0}=\pm 1\}}= & {} \frac{\delta _{l^{'}l-1 }}{\Delta \tau }\left( \begin{array}{ccccccccc} s_{0}(l)\lambda _{0}&{}0&{}0&{}0&{}...&{}0&{}0&{}0&{}0\\ 0&{}-s_{0}(l)\lambda _{0}&{}0&{}0&{}...&{}0&{}0&{}0&{}0\\ 0&{}0&{}s_{0}(l)\lambda _{0}&{}0&{}...&{}0&{}0&{}0&{}0\\ 0&{}0&{}0&{}-s_{0}(l)\lambda _{0}&{}...&{}0&{}0&{}0&{}0\\ \vdots &{}\vdots &{}\vdots &{}\ddots &{}\ddots &{}0&{}0&{}0&{}0\\ 0&{}0&{}0&{}\dots &{}0&{}s_{0}(l)\lambda _{0}&{}0&{}0&{}0\\ 0&{}0&{}0&{}\dots &{}0&{}0&{}-s_{0}(l)\lambda _{0}&{}0&{}0\\ 0&{}0&{}0&{}0&{}0&{}\dots &{}0&{}s_{0}(l)\lambda _{0}&{}0\\ 0&{}0&{}0&{}0&{}0&{}\dots &{}0&{}0&{}-s_{0}(l)\lambda _{0} \end{array}\right) _{2Nc\times 2Nc}\nonumber \\&+(\mu -\frac{u}{2})\mathbf{I}_{2Nc\times 2Nc}, \end{aligned}$$

and $\cosh \lambda _{0} =e^{\frac{1}{2}\Delta \tau u}$. On the sites of the cavity super cell sites Coulomb interaction between electrons does not exist, therefore there is no correlation between spin up and down electrons. Hence all its $\uparrow \downarrow$ and $\downarrow \uparrow$ components are zero

31 $$\begin{aligned} \varvec{{\mathcal {G}}}^{\uparrow \downarrow }_{sc}(i\omega _{n})= & {} \left( \begin{array}{ccccccccc} {{\mathcal {G}}}^{\uparrow \downarrow }_{11}(i\omega _{n})&{}{{\mathcal {G}}}^{\uparrow \downarrow }_{12}(i\omega _{n})&{}...&{}{{\mathcal {G}}}^{\uparrow \downarrow }_{1N_{c}}(i\omega _{n})\\ {{\mathcal {G}}}^{\uparrow \downarrow }_{21}(i\omega _{n})&{}{{\mathcal {G}}}^{\uparrow \downarrow }_{22}(i\omega _{n})&{}...&{}{{\mathcal {G}}}^{\uparrow \downarrow }_{2N_{c}}(i\omega _{n})\\ \vdots &{}\vdots &{}\vdots &{}\vdots \\ {{\mathcal {G}}}^{\uparrow \downarrow }_{N_{c}1}(i\omega _{n})&{}{{\mathcal {G}}}^{\uparrow \downarrow }_{N_{c}2}(i\omega _{n})&{}...&{}{{\mathcal {G}}}^{\uparrow \downarrow }_{N_{c}N_{c}}(i\omega _{n})\\ \end{array}\right) = 0, \end{aligned}$$

and $\varvec{{\mathcal {G}}}^{\downarrow \uparrow }_{sc}(i\omega _{n})=0$. By inserting Eqs. (30) and (31) into Eq. (29) we have $\mathbf{G}^{imp^{\downarrow \uparrow }}_{sc}(i\omega _{n})=\mathbf{G}^{imp^{\uparrow \downarrow }}_{sc}(i\omega _{n})=0$, hence Eq. (29) separates into two equations for spin up and spin down electrons with imaginary time Fourier transform

32 $$\begin{aligned} \mathbf{G}^{imp\;\sigma \sigma }_{sc}(\tau )= \varvec{{\mathcal {G}}}^{\sigma \sigma }_{sc}(\tau )+\int d\tau ^{'}\varvec{{\mathcal {G}}}^{\sigma \sigma }_{sc}(\tau -\tau ^{'}){\mathcal {U}}_{\{s_{0}\}} \mathbf{G}^{imp\;\sigma \sigma }_{sc}(\tau ^{'}), \end{aligned}$$

where

33 $$\begin{aligned} {{\mathcal {U}}}_{\{s_{0}=\pm 1\}}= & {} \frac{\delta _{l^{'}l-1 }}{\Delta \tau }(\delta _{\sigma \uparrow }-\delta _{\sigma \downarrow })\left( \begin{array}{ccccccccc} s_{0}(l)\lambda _{0}&{}0&{}0&{}0&{}...&{}0\\ 0&{}s_{0}(l)\lambda _{0}&{}0&{}0&{}...&{}0\\ 0&{}0&{}s_{0}(l)\lambda _{0}&{}0&{}...&{}0\\ 0&{}0&{}0&{}s_{0}(l)\lambda _{0}&{}...&{}0\\ \vdots &{}\vdots &{}\vdots &{}\ddots &{}\ddots &{}0\\ 0&{}0&{}0&{}\dots &{}0&{}s_{0}(l)\lambda _{0}\\ \end{array}\right) _{Nc\times Nc}+(\mu -\frac{u}{2})\mathbf{I}_{Nc\times Nc}, \end{aligned}$$

in which $\delta _{\sigma \sigma ^{'}}$ is Kronecker delta function.

In the actual calculation of imaginary time discretized of Eq. (32) the following form are using^17^

34 $$\begin{aligned} \left( \mathbf{G}^{imp\;\sigma \sigma }_{sc}(\tau _{l}, \tau _{l^{'}})\right) ^{-1}= \left( \varvec{{\mathcal {G}}}^{\sigma \sigma }_{sc}(\tau _{l},\tau _{l^{'}})\right) ^{-1} e^{\Delta \tau {\mathcal {U}}_{\{s_{0}\}}}+e^{\Delta \tau {\mathcal {U}}_{\{s_{0}\}}}-1, \end{aligned}$$

where $e^{\Delta \tau {\mathcal {U}}_{\{s_{0}\}}}=e^{(\delta _{\sigma \uparrow }-\delta _{\sigma \downarrow })s_{0}\lambda _{0}+(\mu -\frac{u}{2})}\delta _{\tau _{l}\tau _{l^{'}}}$ is a diagonal matrix in imaginary time space. Average over all possible Ising fields configurations of each site in the super cell Green function $\mathbf{G}^{imp\;\sigma \sigma }_{sc}(\tau )$ gives us super cell real space average Green function ${\bar{\mathbf{G}}}^{\sigma \sigma }_{sc}(\tau )$

35 $$\begin{aligned} \langle \mathbf{G}^{imp\;\sigma \sigma }_{sc}(\tau ) \rangle = {\bar{\mathbf{G}}}^{\sigma \sigma }_{sc}(\tau ). \end{aligned}$$

Inverse imaginary Fourier transform of Eq. (35) given by

36 $$\begin{aligned} {\bar{\mathbf{G}}}^{\sigma \sigma }_{sc}(i\omega _{n})=\int d\tau {\bar{\mathbf{G}}}^{\sigma \sigma }_{sc}(\tau ) e^{i\omega _{n}\tau }. \end{aligned}$$

## Real space and k-space self energies and Green functions in the EMSCA

Deriving process of k-space and real space self energy and average Green function EMSCA is as follows. We divide the k-space self energy ${\varvec{\Sigma }}(\mathbf{k}; \tau )$ in to two terms, first term comes from all real space self energies $\Sigma (i,j;\tau )$ that both *i* and *j* sites are inside same super cell and second term including all self energies that *i* and *j* sites are inside different super cells

37 $$\begin{aligned} {\varvec{\Sigma }}(\mathbf{k}; \tau )= & {} \frac{1}{{\mathcal {N}}}\sum _{ij} e^{i\mathbf{k}.\mathbf{r}_{ij}}{\varvec{\Sigma }}(i, j; \tau ), \end{aligned}$$

in which

38 $$\begin{aligned} \mathbf{r}_{i}= & {} \mathbf{r}_{I}+m_{1}{} \mathbf{L}_{c1}+m_{2}\mathbf{L}_{c2}+m_{3}{} \mathbf{L}_{c3};\;\mathbf{r}_{j}=\mathbf{r}_{J}+l_{1}\mathbf{L}_{c1}+l_{2}{} \mathbf{L}_{c2}+l_{3}{} \mathbf{L}_{c3},\nonumber \\ \mathbf{r}_{ij}= & {} \mathbf{r}_{i}-\mathbf{r}_{j}=\mathbf{r}_{IJ}+l\mathbf{L}_{c1}+m\mathbf{L}_{c2}+n\mathbf{L}_{c3}, \end{aligned}$$

where $m_{1},m_{2},m_{3},l_{1},l_{2},l_{3},m,n$ and *l* are integer numbers. $\mathbf{L}_{c1}=N_{c1}{} \mathbf{a}_{1}$, $\mathbf{L}_{c2}=N_{c2}\mathbf{a}_{2}$ and $\mathbf{L}_{c3}=N_{c3}{} \mathbf{a}_{3}$ are super cell lengths as illustrated in Fig. 1. By substitution Eq. (38) in Eq. (37) we have

39 $$\begin{aligned} {\varvec{\Sigma }}(\mathbf{k}; \tau )=\frac{1}{{\mathcal {N}}}\sum _{IJ}\sum _{m,n,l} e^{i\mathbf{k}.\mathbf{r}_{IJ}} e^{i\mathbf{k}.(l\mathbf{L}_{c1}+m\mathbf{L}_{c2}+n\mathbf{L}_{c3})}{\varvec{\Sigma }}(\mathbf{r}_{IJ}+l\mathbf{L}_{c1}+m\mathbf{L}_{c2}+n\mathbf{L}_{c3}; \tau ). \end{aligned}$$

Born–von Karman periodicity condition along lattice lengths implies that

40 $$\begin{aligned} e^{-i\mathbf{k}{{\mathcal {N}}}_{1}.\mathbf{a}_{1}}=\bigg (e^{-i\mathbf{k}N_{c1}.\mathbf{a}_{1}}\bigg )^{\frac{{{\mathcal {N}}}_{1}}{N_{c1}}}=1. \end{aligned}$$

For other lattice lengths we have

41 $$\begin{aligned} e^{-i\mathbf{k}{{\mathcal {N}}}_{2}.\mathbf{a}_{2}}=\bigg (e^{-i\mathbf{k}N_{c2}.\mathbf{a}_{2}}\bigg )^{\frac{{{\mathcal {N}}}_{2}}{N_{c2}}}=1, \end{aligned}$$

and

42 $$\begin{aligned} e^{-i\mathbf{k}{{\mathcal {N}}}_{3}.\mathbf{a}_{3}}=\bigg (e^{-i\mathbf{k}N_{c3}.\mathbf{a}_{3}}\bigg )^{\frac{{{\mathcal {N}}}_{3}}{N_{c3}}}=1. \end{aligned}$$

Since $\frac{{{{\mathcal {N}}}}_{1}}{N_{c1}}$, $\frac{{{{\mathcal {N}}}}_{2}}{N_{c2}}$, $\frac{{{{\mathcal {N}}}}_{3}}{N_{c3}}$ are integer numbers we have

43 $$\begin{aligned} e^{-i\mathbf{k}N_{c1}.\mathbf{a}_{1}}=1,\;\;e^{-i\mathbf{k} N_{c2}.\mathbf{a}_{2}}=1,\;\; e^{-i\mathbf{k}N_{c3}.\mathbf{a}_{3}}=1. \end{aligned}$$

The wave vectors that satisfying Eq. (43) simultaneously are

44 $$\begin{aligned} \mathbf{k}=\frac{n_{1}}{N_{c1}}{} \mathbf{b}_{1}+\frac{n_{2}}{N_{c2}}\mathbf{b}_{2}+\frac{n_{3}}{N_{c3}}{} \mathbf{b}_{3}, \end{aligned}$$

where $n_{1}, n_{2}$ and $n_{3}$ are integer numbers. $\mathbf{b}_{1}, \mathbf{b}_{2}$ and $\mathbf{b}_{3}$ are reciprocal primitive vectors such that $\mathbf{k}$ remains in the FBZ. Number of permitted wave vectors in Eq. (44) are $N_{c}=N_{c1}N_{c2}N_{c3}$. These wave vectors called coarse grain wave vectors and denoted by $\mathbf{k}=\mathbf{K}_{n}$

45 $$\begin{aligned} \mathbf{K}_{n}=\frac{n_{1}}{N_{c1}}{} \mathbf{b}_{1}+\frac{n_{2}}{N_{c2}}\mathbf{b}_{2}+\frac{n_{3}}{N_{c3}}{} \mathbf{b}_{3}. \end{aligned}$$

By real space Fourier transformation of ${\varvec{\Sigma }}(\mathbf{r}_{IJ}+l\mathbf{L}_{c1}+m\mathbf{L}_{c2}+n\mathbf{L}_{c3}; \tau )$ and using Eq. (43) it is easy to show

46 $$\begin{aligned} {\varvec{\Sigma }}(\mathbf{r}_{IJ}+l\mathbf{L}_{c1}+m\mathbf{L}_{c2}+n\mathbf{L}_{c3}; \tau )={\varvec{\Sigma }}(\mathbf{r}_{IJ}; \tau ), \end{aligned}$$

hence Eq. (37) converts to

47 $$\begin{aligned} {\varvec{\Sigma }}(\mathbf{K}_{n};\tau ) =\frac{1}{N_{c}} \sum _{ IJ}e^{i\mathbf{K}_{n}.\mathbf{r}_{IJ}}{\varvec{\Sigma }}_{sc}(I, J; \tau ). \end{aligned}$$

So in the FBZ we have $N_{c}$ different self energies $\{{\varvec{\Sigma }}(\mathbf{K}_{1}; \tau ),...,{\varvec{\Sigma }}(\mathbf{K}_{N_{c}};\tau )\}$. Relation between super cell real space self energy ${\varvec{\Sigma }}_{sc}(I, J; \tau )$ and $\mathbf{K}_{n}$-space ${\varvec{\Sigma }}(\mathbf{K}_{n};\tau )$ is

48 $$\begin{aligned} {\varvec{\Sigma }}_{sc}(I, J; \tau )=\frac{1}{N_{c}} \sum _{ \mathbf{K}_{n}} e^{-i\mathbf{K}_{n}.\mathbf{r}_{IJ}}{\varvec{\Sigma }}(\mathbf{K}_{n};\tau ). \end{aligned}$$

**Figure 2:**
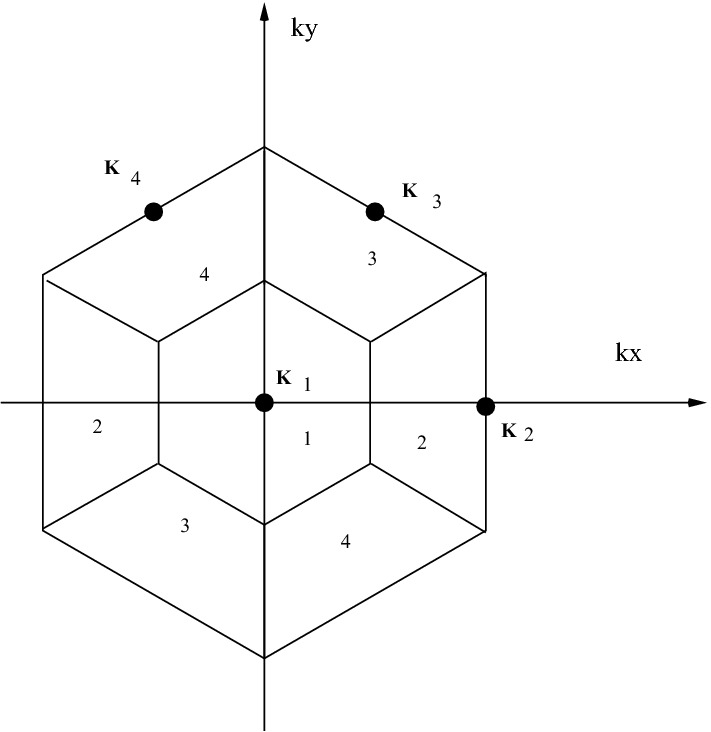
Shows four coarse grain regions of a hexagonal lattice for a four site super cell $N_{c}=4$.

Now by dividing FBZ to $N_{c}$ regions with FBZ symmetry we apply coherent potential approximation formalism to each region that means for all wave vectors $\mathbf{k}$ in the *n*th region self energy values are equal. Figure 2 illustrates four small regions in the graphene first Brillouin zone (FBZ) corresponding to four sites super cell $N_{c}=4$.

For graphene reciprocal primitive vectors are

49 $$\begin{aligned} \mathbf{b}_{1}= \frac{2\pi }{3a_{0}}\mathbf{e}_{x}+\frac{2\pi \sqrt{3}}{3a_{0}}{} \mathbf{e}_{y} \;\;\text{ and }\;\;\mathbf{b}_{2}= \frac{2\pi }{3a_{0}}\mathbf{e}_{x}-\frac{2\pi \sqrt{3}}{3a_{0}}{} \mathbf{e}_{y}, \end{aligned}$$

where $a_{0}$ is nearest neighbors carbon–carbon distance. Hence for this case its coarse Green wave vectors $\mathbf{K}_{n}$ are

50 $$\begin{aligned} \mathbf{K}_{1}=0,\;\mathbf{K}_{2}= \frac{2\pi }{3a_{0}}\mathbf{e}_{x},\;\;\mathbf{K}_{3}= \frac{\pi }{3a_{0}}\mathbf{e}_{x}+\frac{\pi \sqrt{3}}{3a_{0}}{} \mathbf{e}_{y},\;\;\mathbf{K}_{4}= -\frac{\pi }{3a_{0}}{} \mathbf{e}_{x}+\frac{\pi \sqrt{3}}{3a_{0}}{} \mathbf{e}_{y}. \end{aligned}$$

Equation (48) implies that for each $\mathbf{k}=\mathbf{K}_{n}+\mathbf{k}^{'}$ in the *n*th region

51 $$\begin{aligned} e^{i\mathbf{k}^{'}.\mathbf{r}_{IJ}}=1. \end{aligned}$$

By applying Eq. (51) to the Green function we have

52 $$\begin{aligned} \bar{\mathbf{G}}_{sc}(I, J; \tau )=\frac{1}{N_{c}} \sum _{ \mathbf{K}_{n}} e^{-i\mathbf{K}_{n}.\mathbf{r}_{IJ}}\bar{\mathbf{G}}(\mathbf{K}_{n};\tau ),\;\;\bar{\mathbf{G}}(\mathbf{K}_{n};\tau ) =\frac{1}{N_{c}} \sum _{ IJ}e^{i\mathbf{K}_{n}.\mathbf{r}_{IJ}}\bar{\mathbf{G}}_{sc}(I, J; \tau ), \end{aligned}$$

where

53 $$\begin{aligned} \bar{\mathbf{G}}(\mathbf{K}_{n};\tau ) =\frac{N_{c}}{{\mathcal {N}}} \sum _{ \mathbf{k}\in \; nth\; region}\bar{\mathbf{G}}(\mathbf{k}; \tau ). \end{aligned}$$

Algorithm for implementation of method is as follows:

1. A initial guess for self energy usually 0.
2. calculating coarse Green function $\bar{\mathbf{G}}^{\sigma \sigma }_{sc}(\mathbf{K}_{n};i\omega _{m})$ from
  54 $$\begin{aligned} \bar{\mathbf{G}}^{\sigma \sigma }_{sc}(\mathbf{K}_{n};i\omega _{m}) =\frac{N_{c}}{{\mathcal {N}}} \sum _{ \mathbf{k}\in \; nth\; region} \left( (G^{0}(\mathbf{k}; i\omega _{m}))^{-1} -\Sigma ^{\sigma \sigma }_{sc}(\mathbf{K}_{n}; i\omega _{m})\right) ^{-1}. \end{aligned}$$
3. Calculate cavity Green function from $({\mathcal {G}}^{\sigma \sigma }_{sc}(\mathbf{K}_{n}; i\omega _{m}))^{-1}=({{{\bar{G}}}}^{\sigma \sigma }_{sc}(\mathbf{K}_{n}; i\omega _{m}))^{-1}+\Sigma ^{\sigma \sigma }_{sc}(\mathbf{K}_{n}; i\omega _{m})$.
4. Real space and imaginary time Fourier transformation
  55 $$\begin{aligned} {\mathcal {G}}^{\sigma \sigma }_{sc}(I, J; \tau )=\frac{1}{\beta N_{c}}\sum _{\omega _{m}}\sum _{\mathbf{K}_{n}}{\mathcal {G}}^{\sigma \sigma }_{sc}(\mathbf{K}_{n}; i\omega _{m})e^{-i\mathbf{K}_{n}.\mathbf{r}_{IJ}} e^{-i\omega _{m}\tau }. \end{aligned}$$
5. Calculate real space super cell $\mathbf{G}^{imp\;\sigma \sigma }_{sc}(\tau )$ from Eq. (34).
6. Calculate real space super cell average Green function by $\langle \mathbf{G}^{imp\;\sigma \sigma }_{sc}(\tau ) \rangle = {\bar{\mathbf{G}}}^{\sigma \sigma }_{sc}(\tau )$.
7. Calculate $\mathbf{K}_{n}$ and $i\omega _{m}$ Fourier transform of average Green function by
  56 $$\begin{aligned} {{{\bar{G}}}}^{\sigma \sigma }_{sc}(\mathbf{K}_{n}; i\omega _{m})=\int d\tau e^{i\omega _{m}\tau } \frac{1}{Nc}\sum _{I, J} e^{i \mathbf{K}_{n}.\mathbf{r}_{IJ}} {{{\bar{G}}}}^{\sigma \sigma }_{sc}(I,J; \tau ). \end{aligned}$$
8. Calculate new self energy from $\Sigma ^{\sigma \sigma }_{sc}(\mathbf{K}_{n}; i\omega _{m})=({\mathcal {G}}^{\sigma \sigma }_{sc}(\mathbf{K}_{n}; i\omega _{m}))^{-1}-({{\bar{G}}}^{\sigma \sigma }_{sc}(\mathbf{K}_{n}; i\omega _{m}))^{-1}$.
9. Go to step 2 and repeat whole process until convergence.
10. Analytical continuation of self energy to obtain $\Sigma ^{\sigma \sigma }_{sc}(\mathbf{K}_{n}; E+i\eta )$.
11. Calculate $$\Sigma ^{\sigma \sigma }_{sc}(\mathbf{k}; E+i\eta )=\frac{1}{Nc^{2}}\sum _{\mathbf{K}_{n}}\sum _{IJ} e^{i(\mathbf{k}-\mathbf{K}_{n}).\mathbf{r}_{IJ}} \Sigma _{sc}(\mathbf{K}_{n}; E+i\eta )$$.

## Realistic band structure calculation

Process of extracting band structure from calculated ${{{\bar{G}}}}(\mathbf{k}; E+i\eta )$ and $\Sigma (\mathbf{k}; E+i\eta )$ are as follows. The exact k-space single particle effective Green function is

57 $$\begin{aligned} {{{\bar{G}}}}({n\mathbf{k}}; E+i\eta )= & {} \frac{1}{(G^{0}({n\mathbf{k}}; E+i\eta ))^{-1}-\Sigma (\mathbf{k}; E+i\eta )}\nonumber \\= & {} \frac{E-E^{0}_{n\mathbf{k}}-Re\Sigma (\mathbf{k}; E+i\eta )-i(\eta -Im\Sigma (\mathbf{k}; E+i\eta ))}{(E-E^{0}_{n\mathbf{k}}-Re\Sigma (\mathbf{k}; E+i\eta ))^{2}+(\eta -Im\Sigma (\mathbf{k}; E+i\eta ))^{2}}, \end{aligned}$$

where $E^{0}_{n \mathbf{k}}$ are non interacting bands. On the other hand relation between exact effective Green function ${{{\bar{G}}}}({n\mathbf{k}}; E+i\eta )$ and effective band structure $E_{n\mathbf{k}}$ is

58 $$\begin{aligned} {{{\bar{G}}}}({n\mathbf{k}}; E+i\eta )= & {} \frac{1}{E-E_{n\mathbf{k}}+i\eta }=\frac{E-E_{n\mathbf{k}}-i\eta }{(E-E_{n\mathbf{k}})^{2}+\eta ^{2}}. \end{aligned}$$

For an exact effective medium system with whole lattice sites, imaginary part of self energy goes to zero, $lim_{\eta \rightarrow 0}Im\Sigma (\mathbf{k}; E+i\eta )\rightarrow 0$, so the effective band structures $E_{n\mathbf{k}}$ obtain from poles of effective Green function Eq. (57)^16^

59 $$\begin{aligned} E=E_{n\mathbf{k}}=E^{0}_{n\mathbf{k}}+Re\Sigma (\mathbf{k}; E+i\eta ). \end{aligned}$$

In general, effective medium self energy $\Sigma (\mathbf{k}; E+i\eta )$ can not be calculated exactly. Single site dynamical mean field theory (DMFT) with k-independent self energy $\Sigma (\mathbf{k}; E+i\eta )=\Sigma ( E+i\eta )$ is lower approximation. Self energy in the cluster sites approximations such as dynamical cluster approximation (DCA) and effective medium super cell approximation (EMSCA) are step functions, $\Sigma (\mathbf{K}_{m}; E+i\eta )$, that inside each *m* grain in the first Brillouin zone is continuous but at grain boundaries are discontinuous. Although density of states could be calculated from calculated local Green function $N(E)=-\frac{1}{\pi }\ Im {{{\bar{G}}}}(I,I; E+i\eta )$ but self energies discontinuities makes it impossible to calculate renormalized band structure. Another important problem of these approximations is creating fake electronic states which leads to $lim_{\eta \rightarrow 0}Im\Sigma (\mathbf{k}; E+i\eta )\ne 0$. One expect by increasing number of sites in the cluster number of fake states decrease. Relation between *m*th band energy at wave vector $\mathbf{k}$, $E_{m\mathbf{k}}$, and its k-space density of states is

60 $$\begin{aligned} N(m\mathbf{k}; E) =\frac{1}{\pi } \frac{\eta }{(E-E_{m\mathbf{k}})^{2}+\eta ^{2}}, \end{aligned}$$

that has a Dirac delta function feature at $E=E_{m\mathbf{k}}$. One can use this to extract real states eigen values and eliminate fake states. So we should find maximums of calculated density of states, $N(\mathbf{k}; E)=\sum _{n}N(n\mathbf{k}; E)$,

61 $$\begin{aligned} N(\mathbf{k}; E)|_{m\,th \; maximum}= & {} \frac{1}{\pi } \sum _{n} \frac{\eta -Im\Sigma (\mathbf{k}; E+i\eta )}{(E-E^{0}_{n\mathbf{k}}-Re\Sigma (\mathbf{k}; E+i\eta ))^{2}+(\eta -Im\Sigma (\mathbf{k}; E+i\eta ))^{2}}|_{m\,th \; maximum}\nonumber \\\approx & {} \frac{1}{\pi }\frac{\eta }{(E-E_{m\mathbf{k}})^{2}+\eta ^{2}}|_{E=E_{m\mathbf{k}}}. \end{aligned}$$

**Figure 3:**
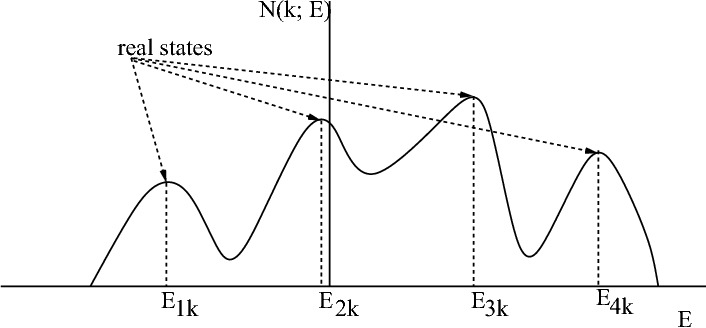
Shows real states eigen values of calculated density of states are maximums of $N(\mathbf{k}; E)$. Other states are fake that should be eliminated.

Figure 3 shows this. To reveal advantage of presented method we calculate renormalized graphene bands by DMFT and four sites, $Nc=4$, beyond super cell approximation for different electrons Coulomb repulsions.

## Results and discussion

**Figure 4:**
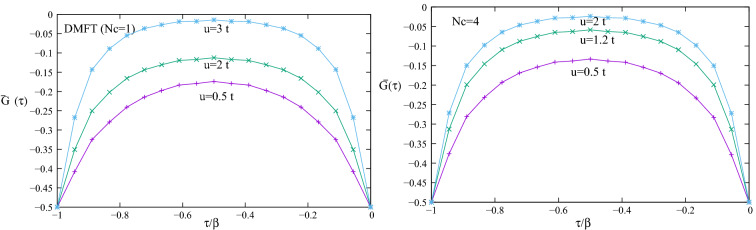
Shows calculated interacting graphene imaginary time average Green function${{\bar{G}}}^{ \uparrow \uparrow }(I,I;\tau )={{\bar{G}}}^{ \downarrow \downarrow }(I,I;\tau )={\bar{G}}(\tau )$ by DMFT $(Nc=1)$ and $Nc=4$ super cell approximation for different electrons Coulomb repulsion at half band filling $n=n_{\uparrow }+n_{\downarrow }=1$, $n_{\uparrow }=0.5$ and $n_{\downarrow }=0.5$.

Now we apply our method to a graphene interacting system with two different cluster sizes $Nc=1$ (DMFT) and $Nc=4$ for different on site electrons repulsions at anti ferromagnetic half band filling $n=n_{\uparrow }+n_{\downarrow }=1$, $n_{\uparrow }=0.5$ and $n_{\downarrow }=0.5$ and $\beta =\frac{1}{0.28 t}$. Note that for this case spin up and down average Green functions are equal ${{\bar{G}}}^{ \uparrow \uparrow }(I,I;\tau )={{\bar{G}}}^{ \downarrow \downarrow }(I,I;\tau )={\bar{G}}(\tau )$. Figure 4 shows calculated interacting graphene imaginary time self consistent average Green function ${\bar{G}}(\tau )$ in terms of $\tau$ for two cluster sites $Nc=1$ and $Nc=4$ for different electrons repulsion interactions. For cluster sites $Nc=4$ the inter sites correlation correction with respect to DMFT is obviously seen. By inserting calculated ${\bar{\mathbf{G}}}(\tau )$ in Eq. (36) and analytical continuation the ${{{\bar{G}}}}(\mathbf{k}; E+i\eta )$ hence $N(\mathbf{k}; E)=-\frac{1}{\pi } Im {{{\bar{G}}}}(\mathbf{k}; E+i\eta )$ obtained. Then energy of each maximum of this calculated density of states $N(\mathbf{k}; E)$ corresponds to one of bands energies.

**Figure 5:**
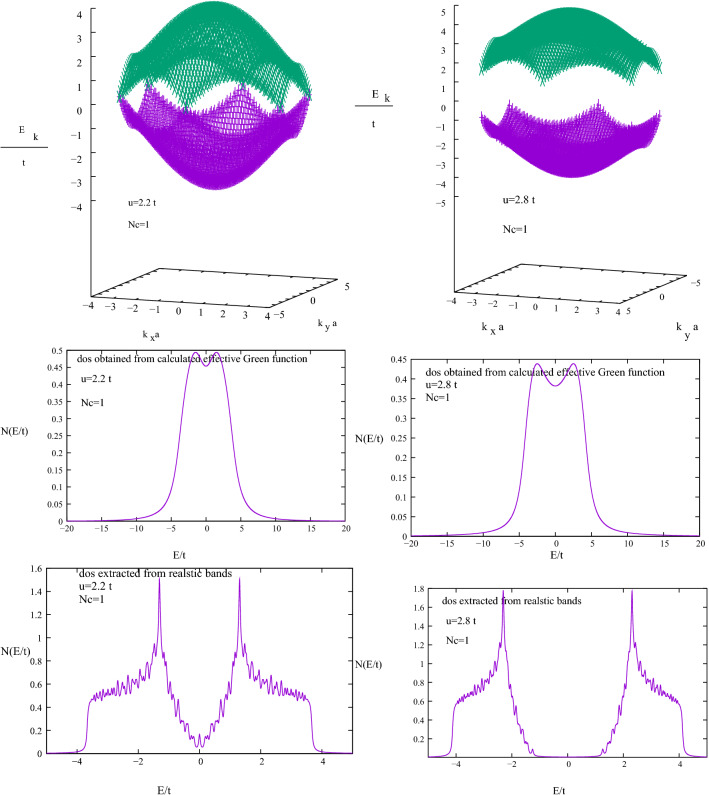
Shows DMFT calculated graphene realistic valance and conduction bands for $u=2.2 t$ and 2.8*t* respectively. The critical repulsion value for an anti ferromagnetic semi metal to an anti ferromagnetic insulator phase transition is $u=2.2 t$. In the $u=2.8 t$ valance and conduction bands are separated but spin up and down bands still are not separated. dos obtained from calculated realistic bands compared with dos obtained directly from DMFT local Green function. We see advantage of our method.

**Figure 6:**
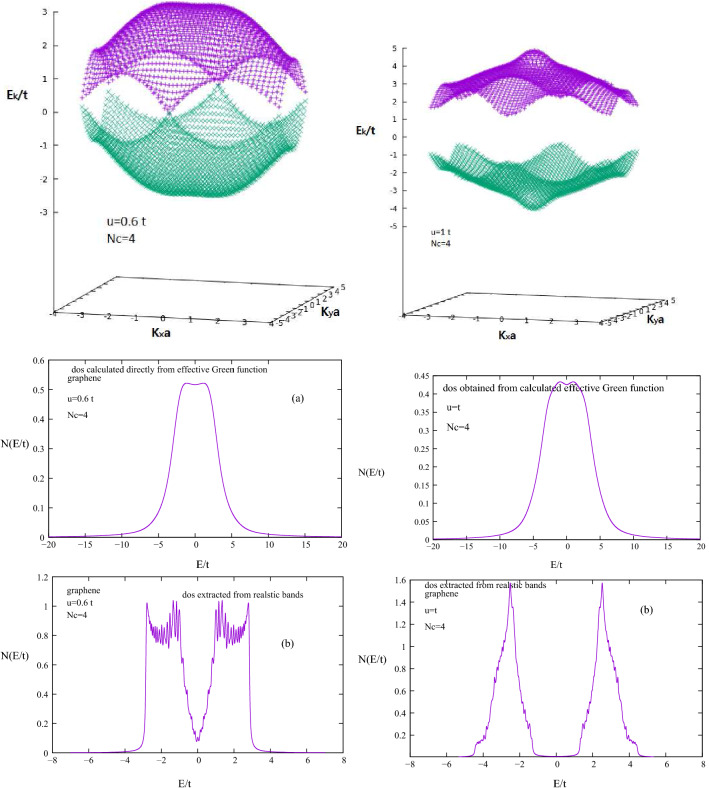
Shows beyond super cell approximation $Nc=4$ calculated realistic bands for repulsion energy $u= 0.6 t, 1 t$. For $u>0.6 t$ a semi metal to anti ferromagnetic phase transition occur. dos obtained from realistic bands with dos obtained from calculated local Green function compared.

First we calculate realistic band structure of this system in the DMFT. Figure 5 shows calculated realistic two bands for $u=2.2 t$ and 2.8*t* in which the fake states are eliminated. To high light advantage of our method we compared the direct DMFT calculated density of states *N*(*E*) with density of states obtained from calculated realistic valance and conduction bands. Our results show that by increasing electrons energy repulsion *u* valance and conduction bands separated but steel valance band is completely full by both spin up and down electrons while conduction band is empty. Our results show that in the DMFT the critical value of repulsion to have a semi metal to an anti ferromagnetic Mott phase transition is $u=2.2 t$. To see effects of multi sites correlation on band structure and density of states for $u=0.6 t$ and 1*t* first we applied four sites $Nc=4$ effective medium super cell approximation to obtain super cell self energy $\Sigma (I, J;E)$ and $\Sigma (\mathbf{K}_{n}; E)$^23–25^. Then we approximate $\mathbf{k}$-space self energy that is continuous in the first Brillouin zone by $\Sigma (\mathbf{k}; E)=\frac{1}{Nc}\sum _{IJ} e^{i \mathbf{k}.\mathbf{r}_{IJ}} \Sigma (I, J; E)$. By substitution this calculated $\Sigma (\mathbf{k}; E)$ in Eq. (61) all bands could be calculated. For these cases Fig. 6 illustrate $Nc=4$ calculated realistic bands and comparison of dos obtained from calculated realistic bands and dos obtained directly from calculated $Nc=4$ super cell local effective Green function $\frac{-1}{\pi }Im {\bar{G}}(I,I;E)$. Our results show that in this repulsion energy spin up and down bands are not separated but for repulsions $u > 0.6 t$ a ferromagnetic semi metal to insulator happened. This is known as anti ferromagnetic Mott insulator phase transition. Comparison of calculated dos from realistic bands and calculated directly from local Green function $\frac{-1}{\pi }Im {\bar{G}}(I,I;E)$ justifies that our method gives us significant correct results.

## Conclusion

For strongly correlated systems we introduced a method for calculation of continues self energy in the whole first Brillouin zone that allows us to calculate renormalized band structure. By using this method the realistic renormalized band structure of Hubbard model of an interacting electrons graphene lattice obtained. Our results show that graphene bands are sensitive to electrons Coulomb repulsion even at low repulsions. Until now people taught that all states obtained by approximated self energies even by DMFT are acceptable but we proved that this method should be corrected to complete band structure calculation process. Our method and results open new perspective on physics of strongly correlated system.
